# Three-Dimensional Speckle-Tracking Echocardiography-Derived Left Ventricular Volumes Show No Associations with Mitral Annular Plane Systolic Excursion in Healthy Adults (Findings from the MAGYAR-Healthy Study)

**DOI:** 10.3390/jcm15103589

**Published:** 2026-05-08

**Authors:** Attila Nemes, Nóra Ambrus, Csaba Lengyel

**Affiliations:** Department of Medicine, Albert Szent-Györgyi Medical School, University of Szeged, 6725 Szeged, Hungary; ambrusnora@gmail.com (N.A.); lengyel.csaba@medu-szeged.hu (C.L.)

**Keywords:** left ventricular, volume, mitral annular plane systolic excursion, three-dimensional, echocardiography, speckle-tracking, healthy

## Abstract

**Introduction.** Left ventricular (LV) function can be characterized by numerous parameters, the most well-known being the ejection fraction (EF), derived from LV end-systolic and end-diastolic volumes (ESV and EDV, respectively) throughout the cardiac cycle. M-mode echocardiography (MME-)-based determination of the longitudinal displacement of the mitral annulus (MA plane systolic excursion, MAPSE) remains a simpler and more practical parameter for assessing LV function in daily clinical routine. Although the relationships between MAPSE, LV deformation and LV rotational mechanics are well-established in healthy individuals, it remains unclear whether MAPSE also correlates with LV volumes themselves. To address this, the associations between LV-ESV and LV-EDV and MAPSE in a healthy cohort were investigated, exploring how these variables relate across average, sub-average, and above-average ranges. **Methods.** The present cohort study enrolled 115 healthy adult volunteers (mean age: 35.3 ± 12.1 years; 68 men). Complete two-dimensional (2D) Doppler echocardiography with MAPSE assessment and 3DSTE-derived measurement of LV volumes was performed in all cases. **Results.** Both end-diastolic and end-systolic LV diameters and volumes tended to be higher in individuals with lower- or higher-than-average MAPSE compared to those with average MAPSE. These differences reached statistical significance for 2D echocardiography-derived LV end-systolic diameter and volume, as well as for 3DSTE-derived LV-EDV and LV-ESV. MAPSE remained consistent regardless of the degree of LV-EDV and LV-ESV. No significant correlations were observed between MAPSE and LV-EDV or LV-ESV. **Conclusions**. LV longitudinal shortening, as represented by MAPSE, is not associated with 3DSTE-derived LV volumes in healthy adults. However, the observed lack of association may not necessarily reflect true physiological independence, but could instead result from limited statistical power, measurement variability, or model misspecification.

## 1. Introduction

Left ventricular (LV) function can be characterized by numerous parameters, the most well-known being the ejection fraction (EF)**,** derived from end-systolic and end-diastolic volumes (ESV and EDV, respectively) throughout the cardiac cycle [1]. Although advanced speckle-tracking echocardiography (STE) enables the assessment of strain parameters, objective measures of global and regional wall contractility [2,3], M-mode echocardiography- (MME-) based determination of the longitudinal displacement of the mitral annulus (MA), i.e., mitral annular plane systolic excursion (MAPSE), remains a simpler and more practical parameter for assessing LV function in daily clinical routine [4,5,6,7,8]. Three-dimensional speckle-tracking echocardiography (3DSTE) enables accurate quantification of LV volumes, a validated approach for which established reference ranges are already available in the literature [9,10,11,12]. Although the relationships between MAPSE, LV deformation, and LV rotational mechanics are well-established in healthy individuals [13,14], it remains unclear whether MAPSE also correlates with LV volumes themselves. To address this, we investigated the associations between LV-ESV, LV-EDV and MAPSE in a healthy cohort, specifically examining these relationships across average, sub-average, and above-average ranges. The study was reported in accordance with the STROBE guidelines.

## 2. Subjects and Methods

**Study population.** The present cohort study enrolled 115 healthy adult volunteers (mean age: 35.3 ± 12.1 years; 68 men) between 2011 and 2017. Participants were recruited via community-based convenience sampling through local advertisements and outreach. The cohort primarily consisted of volunteers from various professional backgrounds, including medical students, academic staff, municipal employees, and law enforcement officers. All participants underwent comprehensive screening, including physical examination, standard 12-lead electrocardiography (ECG), laboratory testing, and conventional two-dimensional (2D) Doppler echocardiography; all results were within normal reference ranges. Exclusion criteria included any known medical conditions or pathological states, regular medication use, smoking, pregnancy, athletic training, or obesity (body mass index > 30 kg/m^2^). This investigation is part of the ‘Motion Analysis of the heart and Great vessels bY three-dimensionAl speckle-tRacking echocardiography in Healthy subjects’ (MAGYAR-Healthy) Study, which aims to establish 3DSTE-derived physiological reference values and explore their clinical associations (‘Magyar’ translates to ‘Hungarian’ in the Hungarian language)(9,13,14). The study was conducted in accordance with the Declaration of Helsinki (as revised in 2013). The protocol was approved by the Institutional and Regional Human Biomedical Research Committee of the University of Szeged, Hungary (registration number: 71/2011; original approval 23 May 2011, latest approval: 17 March 2025). All participants provided written informed consent prior to enrollment.

**Figure 1:**
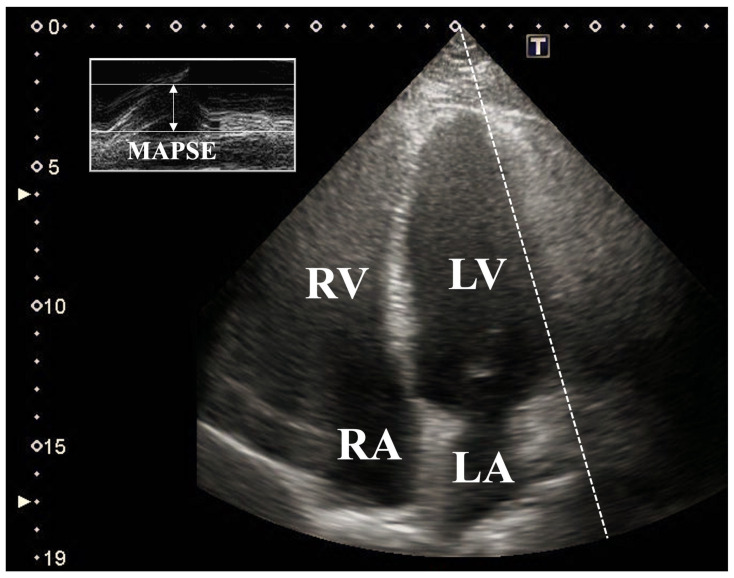
Schematic representation of mitral annular plane systolic excursion (MAPSE) measurement via M-mode echocardiography in the apical four-chamber view. **Abbreviations:** LA = left atrium; LV = left ventricle; RA = right atrium; RV = right ventricle; MAPSE = mitral annular plane systolic excursion.

**MME and 2D Doppler echocardiography.** All echocardiographic evaluations were performed by the same operators using a commercially available Toshiba Artida™ system (Toshiba Medical Systems, Tokyo, Japan) equipped with a PST-30BT (1–5 MHz) phased-array transducer. Healthy subjects were examined in the left lateral decubitus position, with the transducer placed in standard parasternal and apical views. Left atrial (LA) and LV parameters were assessed in accordance with current guidelines, and LV ejection fraction (EF) was calculated using Simpson’s biplane method. Doppler imaging confirmed the absence of valvular regurgitation or stenosis in all subjects [15]. For the assessment of longitudinal LV systolic function, MAPSE was determined using MME in the apical 4-chamber long-axis view, measuring the systolic displacement of the lateral MA edge toward the LV apex [4,5,6,7,8] (Figure 1).

**Figure 2:**
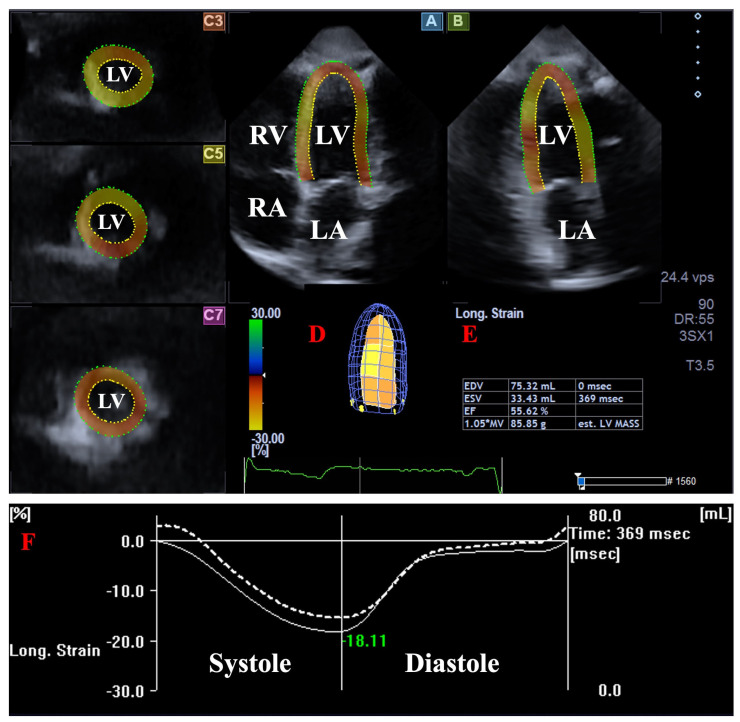
Assessment of left ventricular (LV) volumes using three-dimensional (3D) speckle-tracking echocardiography. The apical longitudinal four-chamber (A) and two-chamber (B) views, along with short-axis views at the apical (C3)**,** mid-ventricular (C5)**,** and basal (C7) LV levels, are displayed alongside a virtual 3D LV cast (D) and the corresponding calculated volumetric data (E). Time–LV volume change curve (dashed white curve) together with time–LV longitudinal strain curve (white curve, not analysed in this study) are presented in (F). **Abbreviations:** LA = left atrium; LV = left ventricle; RA = right atrium; RV = right ventricle; EDV = end-diastolic volume; ESV = end-systolic volume; EF = ejection fraction.

**3DSTE.** 3DSTE was performed in two consecutive steps [16,17,18,19,20]. First, 3D echocardiographic datasets were acquired from the apical window using the same ultrasound system, equipped with a PST-25SX matrix phased-array transducer. To ensure optimal image quality, six cardiac cycle-based subvolumes were captured during a single breath-hold to create a full-volume dataset. In the second step, 3D Wall Motion Tracking software (version 2.7, UltraExtend, Toshiba Medical Systems, Tokyo, Japan) was used for offline analysis. Image quality was qualitatively evaluated based on the clarity of the endocardial border definition across all segments. Datasets exhibiting significant acoustic dropout or stitching artifacts that compromised tracking were excluded. Overall image quality was assessed using a 5-point Likert scale (1: poor to 5: excellent), with a minimum score of 3 required for inclusion in the final analysis. The software automatically generated three short-axis views (basal, mid-ventricular, and apical) and two long-axis views (apical 2-chamber and 4-chamber). The operator manually defined the lateral and septal LV-MA edges and the endocardial surface of the LV apex. Subsequently, automated contour detection and sequential tracking were performed to generate a virtual 3D LV cast. This model was used to determine LV volumes at end-diastole (EDV) and end-systole (ESV), LV-EF and LV mass (Figure 2).

**Statistical analysis.** Data are presented as mean ± standard deviation (SD) or as frequencies and percentages. Variance homogeneity was assessed using Levene’s test, while the Shapiro–Wilk test was used to verify normality of distribution. For continuous variables, independent-samples *t*-tests or Mann–Whitney–Wilcoxon tests were performed for normally and non-normally distributed data, respectively. Multiple comparisons were conducted using one-way analysis of variance (ANOVA) with Bonferroni correction. Correlations were evaluated using Pearson’s and Spearman’s correlation coefficients. Multivariable regression analysis has also been performed. To assess the reproducibility of 3DSTE-derived LV-EDV, LV-ESV, and MAPSE measurements, intra- and interobserver agreement were analyzed in a subset of 25 randomly selected healthy individuals using mean ± 2SD differences and intraclass correlation coefficients (ICCs). Statistical power was retrospectively calculated using G*Power 3.1 (Düsseldorf, Germany), based on the final sample size, a significance level of 0.05, and the observed effect size. A *p*-value < 0.05 was considered statistically significant. All statistical analyses were performed using SPSS software (version 29.0, IBM Corp., Armonk, NY, USA).

## 3. Results

**Clinical characteristics.** Key clinical parameters—including systolic and diastolic blood pressures (122.1 ± 3.2 mmHg and 83.1 ± 2.0 mmHg, respectively), heart rate (71.2 ± 2.2 bpm), height (173.6 ± 934 cm), weight (72.5 ± 13.4 kg), body surface area (1.90 ± 0.21 m^2^) and body mass index (23.9 ± 3.3 kg/m^2^)—were within the normal reference ranges in all healthy subjects.

**Classification of healthy individuals.** The healthy study population was stratified into subgroups based on the mean ± SD of MAPSE and LV-EDV and LV-ESV. Values corresponding to mean—SD (11.2 mm for MAPSE, 63.5 mL for LV-EDV and 25.6 mL for LV-ESV) and mean + SD (17.4 mm for MAPSE, 105.1 mL for LV-EDV and 45.8 mL for LV-ESV) were used as cut-off points to define three distinct subgroups for each parameter (Table 1).

**LV dimensions and volumes in different MAPSE subgroups**. Both end-diastolic and end-systolic LV diameters and volumes tended to be higher in individuals with lower- or higher-than-average MAPSE compared with those with average MAPSE. These differences reached statistical significance for 2D echocardiography-derived LV end-systolic diameter and volume, as well as for 3DSTE-derived LV end-diastolic and end-systolic volumes (Table 1).

**MAPSE in different LV volumes subgroups.** MAPSE remained consistent regardless of the degree of LV-EDV and LV-ESV. Changes in LV dimensions across LV volume groups are presented in Table 2.

**Correlations, multivariable regression and post hoc analyses.** No significant correlations were observed between MAPSE and LV-EDV (r = −0.005, r = 0.965) or LV-ESV (r = −0.077, r = 0.485). After adjusting for key covariates—including age, sex, height, weight, derived body surface area and body mass index, systolic and diastolic blood pressures, heart rate, LV diameters and volumes, MAPSE and LV mass—the results remained consistent with our initial analysis. This suggests that the observed lack of association was not confounded by these factors. A post hoc power analysis was conducted to assess the statistical strength of the observed findings. Based on the current sample size, we identified a large effect size (Cohen’s d = 0.8), with an achieved power of 0.75 at a significance level of α = 0.05.

**Feasibility.** The simultaneous assessment of MME-derived MAPSE and 3DSTE-derived LV volumes was feasible in 115 out of 301 cases, representing a 38% success rate.

**Intraobserver and interobserver agreements.** Table 3 presents the intraobserver and interobserver agreements, including the mean ± 2SD differences and corresponding ICCs for MME-derived MAPSE and 3DSTE-derived LV-EDV and LV-ESV measurements.

## 4. Discussion

During the cardiac cycle, the LV wall undergoes radial thickening, circumferential narrowing, and longitudinal shortening in systole [2,21,22]. Each of these mechanical components can be quantified using STE-derived specific LV strain parameters [2,3]. However, a much simpler method, which has been used in clinical practice for decades, is also available to characterize longitudinal LV function. The method is based on the displacement of the MA toward the cardiac apex during systole, thereby reflecting longitudinal LV function. MAPSE, a well-established and validated parameter characterized by high reproducibility and significant prognostic value, is routinely used in clinical practice to assess longitudinal LV function using conventional, yet easily performed MME [4,5,6,7,8].

3DSTE enables the simultaneous assessment of LV volumes and strain parameters representing LV deformation, as well as features of LV rotational mechanics [13,14,16,17,18,19,20]. Within the framework of the MAGYAR-Healthy Study, relationships between MAPSE, LV strains, and LV rotational mechanics have been previously confirmed in healthy individuals in detail [13,14]. Previous findings suggested that tendentiously reduced LV volumes with preserved LV-EF be observed in individuals with mean MAPSE compared with those with reduced or increased MAPSE [13,14]. The present study is consistent with these findings and further demonstrates that variations in LV-ESV and LV-EDV are not significantly associated with MAPSE. Moreover, no correlations were identified between MAPSE and LV volumes throughout the cardiac cycle. These results can suggest that MA longitudinal shortening is independent of LV volumes. To gain a deeper understanding of the mentioned relationships, future research should focus on large-scale healthy cohorts using high-resolution imaging techniques. Modalities with diagnostic precision matching or surpassing 3DSTE, such as cardiac magnetic resonance, are essential to validate these findings.

**Limitation section.** The most important limitations of the study are presented here:

- Although all enrolled subjects were considered ‘healthy’, the presence of subclinical abnormalities cannot be excluded with absolute certainty. While additional clinical diagnostic testing might have more definitively confirmed their healthy status, performing such examinations in the absence of clinical indications would have raised ethical concerns.
- Moreover, the definition of “healthy” excludes obesity, smoking, and athletic training, which substantially limits external validity and may introduce selection bias toward a highly selected physiologic subset.
- The categorization of continuous variables into mean ± SD subgroups is inherently arbitrary, reduces statistical power, and introduces residual confounding. Furthermore, the use of internal cutoffs lacks clinical relevance and limits interpretability compared to established, guideline-based reference ranges. Future analyses should prioritize continuous modeling or validated clinical thresholds to ensure more robust and actionable findings. These facts should be considered when interpreting results.
- Due to the relatively small healthy cohort, the study may be statistically underpowered, leaving the possibility of Type II errors despite high measurement reproducibility. Consequently, these findings should be considered exploratory and require validation in larger, multi-center studies.
- A limitation of this study is that intra- and interobserver variability analyses were performed in a relatively small cohort of only 25 individuals. While this sample size is consistent with similar 3DSTE studies, a larger population might have provided more robust reproducibility data.
- One of the primary technical limitations of 3DSTE is its relatively low temporal resolution (frame rate: 30 ± 2 fps), which may affect the accuracy of all 3DSTE-derived measurements. Furthermore, 3D-capable matrix transducers are larger than those used for conventional 2D Doppler echocardiography, potentially limiting optimal positioning on the chest wall. Furthermore, the acquisition of six subvolumes over consecutive cardiac cycles may introduce stitching or motion artifacts during data analysis, particularly in cases of inconsistent breath-holding [16,17,18,19,20].
- Although 3DSTE provides a comprehensive volumetric and functional assessment of the LV, including advanced myocardial strain analysis and determination of LV rotational mechanics, the present study did not aim to evaluate associations between MAPSE and these parameters in healthy subjects, as they have already been investigated in detail within the MAGYAR-Healthy Study [13,14].
- While 3D casts can be generated for all cardiac chambers, enabling volumetric and strain analysis of both atria and the right ventricle from the same 3D echocardiographic dataset, such evaluations were beyond the scope of the present study.
- Advanced 3DSTE-based evaluations of the cardiac valves and their annuli, including ‘en-face’ annular measurements and evaluation of ‘sphincter-like’ functional properties, are feasible, but were not aimed to be determined in the present study.
- Direct comparisons between 3DSTE and other advanced imaging modalities, such as 2D Doppler echocardiography for valvular assessment, also remain a subject for future investigations.
- Finally, MAPSE is measured only at the lateral annulus; guideline-recommended approaches typically average septal and lateral values, which may bias estimates and reduce comparability.

**Conclusions**. LV longitudinal shortening, as represented by MAPSE, is not associated with 3DSTE-derived LV volumes in healthy adults. In the presence of lower- or higher-than-average MAPSE, LV dimensions are increased. However, the observed lack of association may not necessarily reflect true physiological independence, but could instead result from limited statistical power, measurement variability, or model misspecification.

## Figures and Tables

**Table 1:** Mitral annular plane systolic excursion and left ventricular volumes in different mitral annular plane systolic excursion subgroups.

	All Subjects (*n* = 115)	MAPSE ≤ 11.2 mm (*n* = 16)	11.2 mm < MAPSE < 17.4 mm (*n* = 79)	17 mm ≤ MAPSE (*n* = 20)
**Two-Dimensional Echocardiography**				
**LA (mm)**	37.4 ± 3.8	38.1 ± 4.5	37.1 ± 3.6	37.9 ± 3.6
**LV-EDD (mm)**	47.9 ± 3.5	49.0 ± 3.7	47.5 ± 3.3	48.6 ± 4.0
**LV-EDV (mL)**	105.8 ± 23.5	109.0 ± 30.2	103.0 ± 20.3	112.8 ± 25.9
**LV-ESD (mm)**	32.0 ± 3.2	33.1 ± 3.8	31.4 ± 2.8 *	32.8 ± 3.5
**LV-ESV (mL)**	37.9 ± 9.1	40.8 ± 9.2	36.5 ± 7.6 *	41.0 ± 12.4 †
**IVS (mm)**	9.2 ± 1.2	9.5 ± 1.4	9.2 ± 1.2	8.8 ± 1.2
**LV-PW (mm)**	9.4 ± 1.5	9.7 ± 1.9	9.3 ± 1.4	9.6 ± 1.4
**LV-EF (%)**	64.5 ± 3.6	64.9 ± 2.7	64.6 ± 3.9	63.9 ± 3.3
**MAPSE (mm)**	14.3 ± 3.1	9.3 ± 1.8	14.2 ± 1.6 *	18.6 ± 1.7 * †
**Three-dimensional speckle-tracking echocardiography**				
**LV-EDV (mL)**	84.3 ± 20.8	95.6 ± 29.1	79.6 ± 19.1 *	89.8 ± 20.5 †
**LV-ESV (mL)**	35.7 ± 10.1	41.3 ± 14.4	34.1 ± 8.4 *	37.5 ± 9.7
**LV-EF (%)**	57.6 ± 5.5	57.1 ± 4.2	57.6 ± 5.8	58.3 ± 5.1
**LV mass (g)**	162.2 ± 32.5	172.1 ± 37.8	159.7 ± 29.2	160.8 ± 37.5

* *p* < 0.05 vs. MAPSE ≤ 11.2 mm, † *p* < 0.05 vs. 11.2 mm < MAPSE < 17.4 mm, statistical significance was set at *p* < 0.017 after Bonferroni correction for three independent comparisons (0.05/3). No results reached this threshold. Abbreviations: LA = left atrium; LV = left ventricular; EDD = end-diastolic diameter; EDV = end-diastolic volume; ESD = end-systolic diameter; ESV = end-systolic volume; IVS = interventricular septum; PW= posterior wall; EF = ejection fraction; MAPSE = mitral annular plane systolic excursion.

**Table 2:** Mitral annular plane systolic excursion and left ventricular volumes in different left ventricular volume subgroups.

	End-Diastolic LV Volume ≤ 63.5 mL (*n* = 16)	63.5 mL ≤ End-Diastolic LV Volume ≤ 105.1 mL (*n* = 84)	End-Diastolic LV Volume ≥ 105.1 mL (*n* = 15)	End-Systolic LV Volume ≤ 25.6 mL (*n* = 14)	25.6 mL ≤ End-Systolic LV Volume ≤ 45.8 mL (*n* = 85)	End-Systolic LV Volume ≥ 45.8 mL (*n* = 16)
**Two-Dimensional Echocardiography**						
**LA (mm)**	36.5 ± 3.8	37.3 ± 3.7	38.9 ± 3.9	37.9 ± 2.6	37.0 ± 3.8	39.0 ± 4.2 &
**LV-EDD (mm)**	46.9 ± 3.1	47.6 ± 3.3	50.8 ± 3.6 * †	48.0 ± 2.7	47.4 ± 3.4	50.7 ± 3.5 ‡ &
**LV-EDV (mL)**	94.6 ± 18.4	104.6 ± 22.6	124.6 ± 22.9 * †	101.9 ± 17.9	103.1 ± 23.1	123.7 ± 22.2 ‡ &
**LV-ESD (mm)**	31.3 ± 3.4	31.6 ± 2.8	34.4 ± 3.8 * †	32.1 ± 2.9	31.6 ± 3.1	33.8 ± 3.1 &
**LV-ESV (mL)**	33.0 ± 6.7	37.4 ± 8.2 *	46.6 ± 11.1 * †	34.4 ± 6.4	37.0 ± 8.4	46.1 ± 10.7 ‡ &
**IVS (mm)**	8.6 ± 1.1	9.2 ± 1.2	10.0 ± 1.0 * †	8.8 ± 1.2	9.1 ± 1.2	10.0 ± 0.9 ‡
**LV-PW (mm)**	9.0 ± 1.3	9.3 ± 1.6	10.0 ± 1.2 *	9.1 ± 1.3	9.3 ± 1.5	9.9 ± 1.3
**LV-EF (%)**	65.7 ± 2.7	64.5 ± 3.7	63.9 ± 3.5	66.3 ± 2.5	64.4 ± 3.8	63.8 ± 3.1 ‡
**MAPSE (mm)**	14.4 ± 3.2	14.3 ± 2.9	14.3 ± 4.1	14.3 ± 3.4	14.2 ± 2.8	14.6 ± 4.1
**Three-dimensional speckle-tracking echocardiography**						
**LV-EDV (mL)**	56.4 ± 5.3	82.7 ± 10.8 *	123.3 ± 16.7 * †	62.2 ± 13.5	81.1 ± 12.4 ‡	120.6 ± 18.3 ‡ &
**LV-ESV (mL)**	24.2 ± 4.7	34.8 ± 6.4 *	53.3 ± 8.5 * †	21.8 ± 2.7	34.7 ± 5.7 ‡	53.5 ± 7.8 ‡ &
**LV-EF (%)**	57.2 ± 6.6	57.8 ± 5.6	56.8 ± 2.6	64.0 ± 5.7	57.0 ± 5.1 ‡	55.5 ± 2.9 ‡
**LV mass (mg)**	131.5 ± 24.1	160.8 ± 27.1 *	203.2 ± 25.7 * †	132.6 ± 22.9	160.5 ± 28.3 ‡	197.2 ± 29.3 ‡ &

* *p* < 0.05 vs. end-diastolic LV volume ≤ 63.5 mL; † *p* < 0.05 vs. 63.5 mL ≤ end-diastolic LV volume ≤ 105.1 mL; ‡ *p* < 0.05 vs. end-systolic LV volume ≤ 25.6 mL; & *p* < 0.05 vs. 25.6 mL ≤ end-systolic LV volume ≤ 45.8 mL, statistical significance was set at *p* < 0.017 after Bonferroni correction for three independent comparisons (0.05/3). No results reached this threshold. Abbreviations: LA = left atrium; LV = left ventricular; EDD = end-diastolic diameter; EDV = end-diastolic volume; ESD = end-systolic diameter; ESV = end-systolic volume; IVS = interventricular septum; PW = posterior wall; EF = ejection fraction; MAPSE = mitral annular plane systolic excursion.

**Table 3:** Intra- and interobserver variability for mitral annular plane systolic excursion and left ventricular volumes as assessed by three-dimensional speckle-tracking echocardiography.

	Intraobserver Agreement		Interobserver Agreement	
	Mean ± 2SD for Values Obtained from Duplicate Measurements by the Same Observer	ICC Between Measurements of the Same Observer	Mean Difference ± 2SD Between Measurements Obtained by Two Independent Observers	ICC Between Independent Measurements of 2 Observers
**MAPSE (mm)**	0.52 ± 0.21	0.95 (*p* < 0.01)	0.54 ± 0.18	0.96 (*p* < 0.01)
**LV-EDV (mL)**	1.56 ± 5.82	0.90 (*p* < 0.01)	1.61 ± 4.87	0.91 (*p* < 0.01)
**LV-ESV (mL)**	0.69 ± 4.35	0.91 (*p* < 0.01)	0.88 ± 4.06	0.92 (*p* < 0.01)

Abbreviations. SD = standard deviation; ICC = intraclass correlation coefficient; MAPSE = mitral annular plane systolic excursion; LV = left ventricular; EDV = end-diastolic volume; ESV = end-systolic volume.

## Data Availability

The original contributions presented in the study are included in the article; further inquiries can be directed to the corresponding authors.
